# Transcriptomic Profile of *Mycobacterium smegmatis* in Response to an Imidazo[1,2-*b*][1,2,4,5]tetrazine Reveals Its Possible Impact on Iron Metabolism

**DOI:** 10.3389/fmicb.2021.724042

**Published:** 2021-08-04

**Authors:** Aleksey A. Vatlin, Egor A. Shitikov, Mohd Shahbaaz, Dmitry A. Bespiatykh, Ksenia M. Klimina, Alan Christoffels, Valery N. Danilenko, Dmitry A. Maslov

**Affiliations:** ^1^Laboratory of Bacterial Genetics, Vavilov Institute of General Genetics Russian Academy of Sciences, Moscow, Russia; ^2^Peoples’ Friendship University of Russia (RUDN University), Moscow, Russia; ^3^Department of Molecular Biology and Genetics, Federal Research and Clinical Center of Physical-Chemical Medicine of Federal Medical Biological Agency, Moscow, Russia; ^4^South Africa Medical Research Council Bioinformatics Unit, South African National Bioinformatics Institute, University of the Western Cape, Cape Town, South Africa

**Keywords:** *Mycobacterium*, drug resistance, imidazo[1, 2-*b*][1, 2, 4, 5]tetrazine, transcriptome, drug development, tuberculosis, virtual screening

## Abstract

Tuberculosis (TB), caused by the *Mycobacterium tuberculosis* complex bacteria, is one of the most pressing health problems. The development of new drugs and new therapeutic regimens effective against the pathogen is one of the greatest challenges in the way of tuberculosis control. Imidazo[1,2-*b*][1,2,4,5]tetrazines have shown promising activity against *M. tuberculosis* and *M. smegmatis* strains. Mutations in *MSMEG_1380* lead to *mmpS5–mmpL5* operon overexpression, which provides *M. smegmatis* with efflux-mediated resistance to imidazo[1,2-*b*][1,2,4,5]tetrazines, but the exact mechanism of action of these compounds remains unknown. To assess the mode of action of imidazo[1,2-*b*][1,2,4,5]tetrazines, we analyzed the transcriptomic response of *M. smegmatis* to three different concentrations of **3a** compound: 1/8×, 1/4×, and 1/2× MIC. Six groups of genes responsible for siderophore synthesis and transport were upregulated in a dose-dependent manner, while virtual docking revealed proteins involved in siderophore synthesis as possible targets for **3a**. Thus, we suggest that imidazo[1,2-*b*][1,2,4,5]tetrazines may affect mycobacterial iron metabolism.

## Introduction

*Mycobacterium tuberculosis*, the causative agent of tuberculosis (TB), is one of the most successful bacterial pathogens. Despite many attempts to control the disease, it remains one of the world’s major health problems that causes more than 1.4 million deaths annually. The situation is complicated by the emergence of drug-resistant forms of the disease [World Health Organization (WHO)., 2020]. And if the initial mathematical models suggested that resistant isolates should be less transmissible and therefore unlikely to spread successfully in human populations, now it is clear that compensatory evolution and other factors drive the successful distribution of multidrug-resistant tuberculosis (Gagneux, 2009). In turn, infection with such isolates leads to longer treatment time with more toxic and costlier than the first-line drugs-based regimens and low treatment success (Rajbhandary et al., 2004). Hence there is an urgent need for novel drugs that are active against *M. tuberculosis*.

We have previously described imidazo[1,2-*b*][1,2,4,5]tetrazines as a class of promising antimycobacterial agents, with relatively high efficacy against both *M. tuberculosis* and *M. smegmatis*. An original test-system *M. smegmatis aphVIII*+ and docking studies showed that eukaryotic type serine-threonine protein kinases (STPKs) may act as targets of these compounds (Maslov et al., 2017, 2019). The main mechanism of resistance to these compounds is provided by mutations in *MSMEG_1380*, a transcriptional repressor of the *mmpS5–mmpL5* efflux system. These mutations result in *mmpS5–mmpL5* overexpression and increased efflux of the compounds from the cell (Maslov et al., 2020). A similar mechanism of resistance was also shown for bedaquiline and clofazimine (Hartkoorn et al., 2014), azoles (Milano et al., 2009), thiacetazone derivatives (Halloum et al., 2017), and tryptanthrins (Frolova et al., 2021) in various mycobacterial species. However, the mode of action, as well as biotarget(s) of imidazo[1,2-*b*][1,2,4,5]tetrazines have not yet been confirmed *in vitro*.

Additionally, we have constructed an *M. smegmatis mc2 155* mutant, carrying a 2828 bp deletion in the *mmpS5–mmpL5* operon, namely *M. smegmatis* Δ*mmp5*, which was hypersensitive to imidazo[1,2-*b*][1,2,4,5]tetrazines (Shur et al., 2021). We used this strain in an attempt to obtain spontaneous mutants, resistant to the imidazo[1,2-*b*][1,2,4,5]tetrazines, with a mechanism different from *mmpS5–mmpL5* overexpression, but could not do it even at a frequency of 10^–10^. This fact may indicate that these compounds have more than one biotarget, making the standard genetic approach useless in this case.

Transcriptomic studies, consisting of the analysis of the total RNA sequencing (RNA-seq), are now becoming a powerful tool for establishing additional mechanisms of action of antimicrobial agents, including anti-TB drugs, through the bacterial transcriptional response in the presence of the drug. This approach was used to establish the mechanisms of action of bedaquiline on dormant *M. tuberculosis* cells (Hards et al., 2015), mechanisms of ethionamide resistance of XDR-TB clinical isolates (Welzen et al., 2017), and to other antibiotics used in clinical practice (Briffotaux et al., 2019).

In the initial study we have tried to analyze the transcriptomic response of *M. smegmatis* to one of the imidazo[1,2-*b*][1,2,4,5]tetrazines – **3a** (Supplementary Figure 1), treating the cells with 2× MIC (256 μg/ml) for 90 min, but this has led to a differential expression of over 1300 genes, making a detailed analysis rather intricate (Vatlin et al., 2020).

In this work, we describe the transcriptomic analysis of *M. smegmatis* in the presence of different subinhibitory concentrations of the imidazo[1,2-*b*][1,2,4,5]tetrazine **3a** (1/8×, 1/4×, and 1/2× MIC) to get an insight in the gradual changes in the bacterial response to the increasing amounts of this drug. We have observed a dose-dependent differential expression of genes involved in iron acquisition, transport and storage, as well as other iron-regulated genes, showing a state of iron-starvation induced by **3a**. In turn further virtual docking studies revealed siderophore synthesis proteins as potential targets of **3a**.

## Materials and Methods

### Bacterial Strain and Growth Conditions

*Mycobacterium smegmatis mc2 155* was grown in Middlebrook 7H9 medium (Himedia, India) supplemented with oleic albumin dextrose catalase (OADC, Himedia, India), 0.1% Tween-80 (v/v), and 0.4% glycerol (v/v), while soyabean-casein digest agar (M290, Himedia, India) and Middlebrook 7H11 agar (Himedia, India) supplemented with OADC were used as the solid media. Bacterial cultures in liquid medium were incubated in the Multitron incubator shaker (Infors HT, Basel, Switzerland) at 37°C and 250 rpm.

For drug exposure assay and transcriptomic analysis, *M. smegmatis mc2 155* was inoculated from the agarized plates in 7H9 medium and grown until OD_600_ = 2.5 (two nights) to obtain a stable liquid culture without clumps, diluted in the proportion 1:200 and cultured overnight until OD_600_ = 2, then diluted in the proportion of 1:10 in fresh medium (to an approximate OD_600_ = 0.2). **3a** 100× stocks were prepared in DMSO and added to the bacterial cultures to a final concentration corresponding to 1/8× MIC (16 μg/ml), 1/4× MIC (32 μg/ml), and 1/2× MIC (64 μg/ml) in 7H9 OADC medium (Maslov et al., 2020). The same volume of DMSO was added to the control samples (1% v/v). Bacterial cultures were incubated for 90 min [1/2 of the cell division time (Logsdon and Aldridge, 2018)] at 37°C and 250 rpm and proceeded to RNA extraction. The experiments were carried in three biological replicates.

The paper-disk drug susceptibility assay was performed as described before (Maslov et al., 2019). Briefly, *M. smegmatis mc2 155* was grown in Middlebrook 7H9 broth to midexponential phase (OD_600_ = 1.2). Afterwards, the culture was diluted in the proportion of 1:9:10 (culture:water:M290 medium) and 5 ml were poured as the top layer on Petri dishes with agarized M290 medium. The plates were allowed to dry for at least 30 min, afterwards sterile paper disks with impregnated 300 nmol of **3a** were plated. The plates were incubated for 2–3 days at 37°C, until the bacterial lawn was fully grown. Growth inhibition halos were measured to the nearest 1 mm. The experiments were carried out as triplicates; the average diameter and standard deviation (SD) were calculated. Those differences that had no intersection of the SDs with the control were considered significant.

### Total RNA Extraction

Cells from 10 ml culture were harvested by centrifugation for 10 min at 3000 × *g* and 4°C, washed twice by 10 ml of fresh Middlebrook 7H9 broth to remove the traces of **3a** and DMSO and once by 3 ml of RNAprotect Bacteria Reagent (Qiagen, United States) for RNA stabilization. *M. smegmatis* cells were homogenized in ExtractRNA reagent (Evrogen, Russia), followed by phenol (pH = 4.5)-chloroform-isoamyl alcohol (25:24:1) purification and precipitation with isopropanol (2:1, v/v).

DNase treatment was carried out as previously described (Bespyatykh et al., 2019) with TURBO DNA-free kit (Thermo Fisher Scientific, Waltham, MA, United States), in volumes of 100 μl, and further with the RNase-Free DNase Set (Qiagen, Hilden, Germany), according to the manufacturers’ protocol. RNA cleanup was performed with the RNeasy Mini Kit (Qiagen). The concentration and quality of the total RNA were checked by the Quant-it RiboGreen RNA assay (Thermo Fisher Scientific) and the RNA 6000 Pico chip (Agilent Technologies, Santa Clara, CA, United States), respectively.

### Library Preparation and RNA Sequencing

Total RNA (1 μg) was used for library preparation as previously described (Bespyatykh et al., 2020) with some modifications. Ribosomal RNA was removed from the total RNA using the Ribo-Zero Plus rRNA Depletion Kit (Illumina, United States) and libraries were prepared using the NEBNext Ultra II Directional RNA Library Prep Kit (NEB), according to the manufacturer’s protocol. Subsequently, RNA cleanup was performed with the Agencourt RNA Clean XP kit (Beckman Coulter, Brea, United States). The library underwent a final cleanup using the Agencourt AMPure XP system (Beckman Coulter) after which the libraries’ size distribution and quality were assessed using a high sensitivity DNA chip (Agilent Technologies). Libraries were subsequently quantified by Quant-iT DNA Assay Kit, High Sensitivity (Thermo Fisher Scientific). Finally, equimolar quantities of all libraries (12 pM) were sequenced by a high throughput run on the Illumina HiSeq using 2 × 100 bp paired-end reads and a 5% Phix spike-in control. RNA-seq read data were deposited to the NCBI Sequence Read Archive under accession number PRJNA615922.

### Bioinformatics Analysis

Quality control of the raw sequencing data was performed using FastQC (v.0.11.9) (Andrews, 2010), individual reports were merged with MultiQC (v.1.9) (Ewels et al., 2016). Adapters and low-quality reads were removed using Trimmomatic (v.0.39) (Bolger et al., 2014). HISAT2 (v.2.2.1) (Kim et al., 2019) was used to map trimmed reads to the reference *M. smegmatis mc2 155* genome (CP000480.1). Mapping quality and coverage along genes were assessed with QualiMap (v.2.2.2) (Okonechnikov et al., 2016). Mapped reads were assigned to genes with featureCounts (v.2.0.1) (Liao et al., 2014). Differential gene expression (DGE) analysis was performed using the edgeR (v.3.30.3) (Robinson et al., 2010) for R (v.4.0.2) (R Core Team, 2020). Genes with a false discovery rate (FDR) cutoff of 0.001 and fold change (FC) log_2_FC ≥ 1, or log_2_FC ≤ −1 were considered to be differentially expressed. Plots were generated within R (v.4.0.2) using ggplot2 (v.3.3.2) (Wickham, 2009) and cowplot (v.1.1.0) (Wilke, 2016) packages. Further functional enrichment analysis of the COG categories and Kyoto Encyclopedia of Genes and Genomes (KEGG) pathways for differentially expressed genes (DEGs) was performed using the DAVID (v.6.8) (Huang et al., 2009) database, categories were considered enriched with *p* ≤ 0.05.

### Virtual Screening Studies

To understand the effect of the imidazo[1,2-*b*][1,2,4,5]tetrazine on the structures of proteins associated with the iron metabolism in *M. smegmatis*, the molecular docking was performed using AutoDock vina (Trott and Olson, 2010). Primarily, the sequence information regarding the listed protein was obtained from the UniProt.^1^ As the majority of proteins don’t have X-ray crystallographic (X-ray) structures available in the biological database, therefore, the SWISS-MODEL was used which is very efficient in modeling proteins with amino acid numbers as large as 5000 (Schwede et al., 2003). The 3-D coordinates of the respective proteins were prepared and optimized using the utilities of MAESTRO (Schrödinger Release 2018-1: Maestro, Schrödinger, LLC, New York, NY, United States, 2018) and the obtained structures were energetically minimized using the modules present in JAGUAR (Bochevarov et al., 2013). Consequently, the docking was performed using AutoDock vina and docked conformations were predicted through the combination of free energy functionals, empirical force field and the Lamarckian Genetic Algorithm (Trott and Olson, 2010). The dimensional space of 40 × 40 × 40 Å was set along the XYZ directions with varied central coordinates and the spacing was set to the 0.375 Å. To achieve the maximum accuracy, the parameters were set to the highest efficiency range.

## Results

### Transcriptional Response of *M. smegmatis* to 3a Treatment – KEGG Analysis

To evaluate the changes in bacterial metabolism during **3a** treatment, the transcriptomic profiles of *M. smegmatis mc2 155* strain exposed to **3a** at 1/8×, 1/4×, and 1/2× MIC for a duration of 90 min were examined. In total, approximately 6000 quantifiable transcripts were identified for each experiment. These results are comparable to the RNA-seq of the DMSO-treated cells (control) and correspond to almost complete coverage of the genome. A non-metric multidimensional scaling analysis showed a clear separation between samples exposed to different **3a** concentrations (Supplementary Figure 2).

Analysis of DEGs revealed that **3a** significantly (log_2_FC ≥ 1, or log_2_FC ≤ −1, FDR < 0.001) variates the expression level of numerous genes. We observed that 7.4% (452/6079) genes were upregulated and 14.1% (856/6079) were downregulated upon exposure to 16 μg/ml **3a**, with these numbers increasing up to 13.9 and 14.9%, respectively, at the exposure to 64 μg/ml **3a**, as compared to the control (Supplementary Table 1). We were able to detect a dose-dependent effect (at least a twofold increase or decrease of expression FC, upon twofold increase in **3a** concentration) for 120 and 173 genes, respectively (Supplementary Table 2). Upon implementation of an additional filter (at least a 2× FC at 1/4× MIC relative to 1/8× MIC, and at least a 2× FC at 1/2× MIC relative to 1/4× MIC) we were left with just 31 overexpressed and 11 underexpressed genes (Supplementary Table 3). Among those 31 overexpressed genes, 25 were related to iron acquisition and transport (exochelin synthesis and transport, *mbt-1* and *mbt-2* clusters, ESX-3 operon and a gene encoding a siderophore-interacting protein).

**FIGURE 1:**
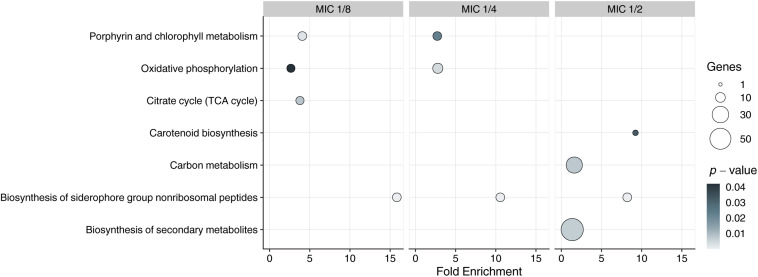
Kyoto Encyclopedia ofGenes and Genomes enrichment analysis of DEGs in exposed to **3a** samples with different MICs. *Y*-axis represents KEGG pathways, *X*-axis represents fold enrichment (amount of input pathway DEGs/background gene set, ≥1.5 considered as interesting). Dots colors represent the *p*-values of enrichment, the dots’ size represents the gene number in the pathway.

DAVID functional annotation tool was used to infer enriched KEGG pathways from sets of upregulated DEGs. Enriched pathway (*p* < 0.05) that was present in all MICs was “biosynthesis of siderophore group non-ribosomal peptides,” and comprised eight genes (*MSMEG_4509*–*MSMEG_4513*, *MSMEG_4515*, *MSMEG_4516*, and *MSMEG_4524*) in all concentrations of the compound (Figure 1).

### Dose-Dependent Gene Regulation Reveals Further Impact of Imidazo[1,2-*b*][1,2,4,5]tetrazine on Iron Metabolism

**FIGURE 2:**
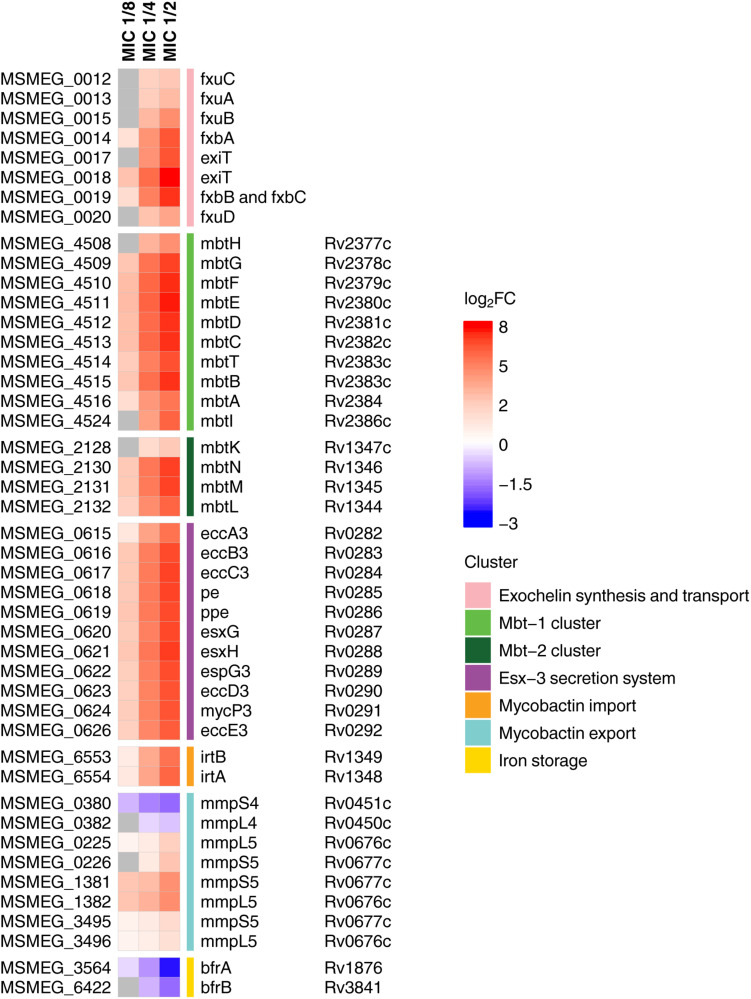
Heatmap showing log_2_-transformed fold change in expression levels of iron metabolites during **3a** exposure. *M. smegmatis mc2 155* locus tags (MSMEG_*) are indicated to the left of the heatmap, and *M. tuberculosis* H37Rv locus tags (Rv*) are indicated to the right of the heatmap, genes’ names are relative to *M. smegmatis mc2 155*. Genes with FDR > 0.001 are shown in gray.

Since the siderophore biosynthesis KEGG pathway was enriched in response to **3a** exposure (Figure 1), we have decided to conduct a deeper analysis of the genes involved in the iron metabolism (Figure 2), using their homologs in *M. tuberculosis* H37Rv according to Namouchi et al. (2017) as an additional reference, as more data is published for this organism.

We have found that in compliance with the KEGG analysis, the *mbt* gene clusters were upregulated in a dose-dependent manner (Figure 2). These operons are the key elements of the “biosynthesis of siderophore group non-ribosomal peptides” pathway, which is responsible for the synthesis of the most important iron-chelating compounds – siderophores (mycobactin and carboxymycobactin in mycobacteria) (Chao et al., 2018). Similar changes were observed for the biosynthesis of exochelin, which is specific for non-virulent mycobacteria (Ratledge and Ewing, 1996). Opposite changes have been shown in iron storage genes *bfrA* and *bfrB* (Reddy et al., 2012), expression of which declined with increasing dose of the compound.

Overexpression of genes involved in iron and siderophore acquisition and transport was also observed (Figure 2). Among these overexpressed genes was ESX-3 operon, which is a vital core operon and is part of the type VII secretion system (T7SS), responsible for iron acquisition and controlled by the iron-dependent transcriptional repressor (IdeR) (Ioerger et al., 2013). The iron-dependent transporters *irtA* and *irtB* (Ryndak et al., 2010), and the genes of the *mmpS5–mmpL5* siderophore transport system (Wells et al., 2013) were also amidst overexpressed genes, though *mmpS4–mmpL4* genes were downregulated.

### *In silico* Interaction Studies Reveal Siderophore Synthesis Genes as Potential 3a Targets

As the transcriptomic analysis revealed the overexpression of iron acquisition and transport genes in response to **3a** exposure, with the highest impact observed for siderophore synthesis genes. Therefore, to understand the structural complementarity of **3a** with the selected 46 overexpressed genes, an extensive molecular docking study was performed, which revealed the binding preferences of the inhibitor molecule. Consequently, streamlined filtering enabled the selection of specific protein targets such as protein FxuA which is considered to be significant in the biosynthesis of exochelin, one of the two siderophores present in *M. smegmatis* which are involved in iron transport (Fiss et al., 1994). Similarly, FxbA which was identified to be a putative formyltransferase catalyzed the addition of the formyl group in the exochelin biosynthesis (Fiss et al., 1994). Through docking studies, it was observed that the imidazo[1,2-*b*][1,2,4,5]tetrazine was bonded with the highest affinity to the structure of FxuA followed by FxbA with free energies of interaction −8.0 and −7.8 kcal/mol, respectively, among the 46 docked systems (Table 1). These observations showed that the activity of exochelin could be inhibited to a relatively higher extent by the administration of imidazo[1,2-*b*][1,2,4,5]tetrazine. The compound was observed to be interacting with Ser41, Thr42, Trp68, Arg69, Arg72, and Leu309 of FxuA, while for FxbA the interactions were observed with Ala85, Asn87, Trp88, Thr90, Asn106, His108, Phe118, and Leu143 (Supplementary Figures 3, 4). The output of MetaPocket (Huang, 2009) revealed that the respective interacting residues form the part of ligand binding cavities. Furthermore, among the mycobactin biosynthesis enzymes, the highest binding affinities were observed with MbtC and MbtD with calculated free energies of −7.7 kcal/mol (Table 1). The MbtC binding pocket contains Pro282, His311, Thr313, Gly318, Phe415, and Met417, while for MbtD the amino acids Gln104, His190, Ala262, Ile287, and Val293 were observed in the interaction pocket (Supplementary Figures 5, 6). Similar binding affinity was observed for FxbC with the free energy of −7.7 kcal/mol and interacting residues are Ser1718, Phe1762, Phe1764, Ile1863, Thr1864, and His2004 (Supplementary Figure 7). Moreover, a relatively higher binding affinity of −7.8 kcal/mol was observed for EccA3 protein which was characterized to be a member of CbbX family ATPases involved in the ESX-3 secretory pathway (Gaur et al., 2017). We observed that the EccA3 contains the residues Pro366, Glu542, Arg543, Ser545, Ala549, Tyr648, and Arg469 in the docked pocket of the protein (Supplementary Figure 8).

**TABLE 1:** List of differential inhibitory efficiency in the form of free energy of binding for against the proteins involved in iron acquisition and transport in *M. smegmatis*.

S. No	*M. smegmatis* locus tag	Protein symbol	Free energy (kcal/mol)

Exochelin synthesis and transport			
1	MSMEG_0012	FxuC	−6.9
2	MSMEG_0013	FxuA	−8.0
3	MSMEG_0015	FxuB	−6.3
4	MSMEG_0014	FxbA	−7.8
5	MSMEG_0017	ExiT	−6.6
6	MSMEG_0018	ExiT	−6.8
7	MSMEG_0019	FxbB	−7.0
8	MSMEG_0019	FxbC	−7.7
9	MSMEG_0020	FxuD	−5.7

**Mbt-1 cluster**			

10	MSMEG_4508	MbtH	−5.4
11	MSMEG_4509	MbtG	−7.2
12	MSMEG_4510	MbtF	−7.1
13	MSMEG_4511	MbtE	−7.6
14	MSMEG_4512	MbtD	−7.7
15	MSMEG_4513	MbtC	−7.7
16	MSMEG_4514	MbtT	−6.6
17	MSMEG_4515	MbtB	−7.2
18	MSMEG_4516	MbtA	−7.6
19	MSMEG_4524	MbtI	−6.8

**Mbt-2 cluster**			

20	MSMEG_2128	MbtK	−7.4
21	MSMEG_2130	MbtN	−7.1
22	MSMEG_2131	MbtM	−7.0
23	MSMEG_2132	MbtL	−5.7

**Esx-3 secretion system**			

24	MSMEG_0615	EccA3	−7.8
25	MSMEG_0616	EccB3	−5.4
26	MSMEG_0617	EccC3	−6.9
27	MSMEG_0618	PE	−5.7
28	MSMEG_0619	PPE	−5.8
29	MSMEG_0620	EsxG	−6.1
30	MSMEG_0621	EsxH	−5.1
31	MSMEG_0622	EspG3	−6.8
32	MSMEG_0623	EccD3	−6.5
33	MSMEG_0624	MycP3	−6.9
34	MSMEG_0626	EccE3	−6.5

**Mycobactin import**			

35	MSMEG_6553	IrtB	−6.5
36	MSMEG_6554	IrtA	−6.8

**Mycobactin export**			

37	MSMEG_0380	MmpS4	−6.0
38	MSMEG_0382	MmpL4	−7.1
39	MSMEG_0225	MmpL5	−7.2
40	MSMEG_0226	MmpS5	−6.8
41	MSMEG_1381	MmpS5	−6.3
42	MSMEG_1382	MmpL5	−6.7
43	MSMEG_3495	MmpS5	−6.6
44	MSMEG_3496	MmpL5	−7.5

**Iron storage**			

45	MSMEG_3564	BfrA	−5.9
46	MSMEG_6422	BfrB	−6.0

### Addition of Iron to the Growth Medium Reduces *M. smegmatis* 3a Susceptibility

We have previously observed that *M. smegmatis* had different **3a** MIC values in 7H9 + OADC and Lemco-Tw medium (128 and 64 μg/ml, respectively) (Maslov et al., 2019, 2020). With our findings, that **3a** could lead to iron starvation by inhibiting siderophore synthesis, this could be due to the additional iron in the composition of Middlebrook 7H9 broth in the form of ferric ammonium citrate.

**FIGURE 3:**
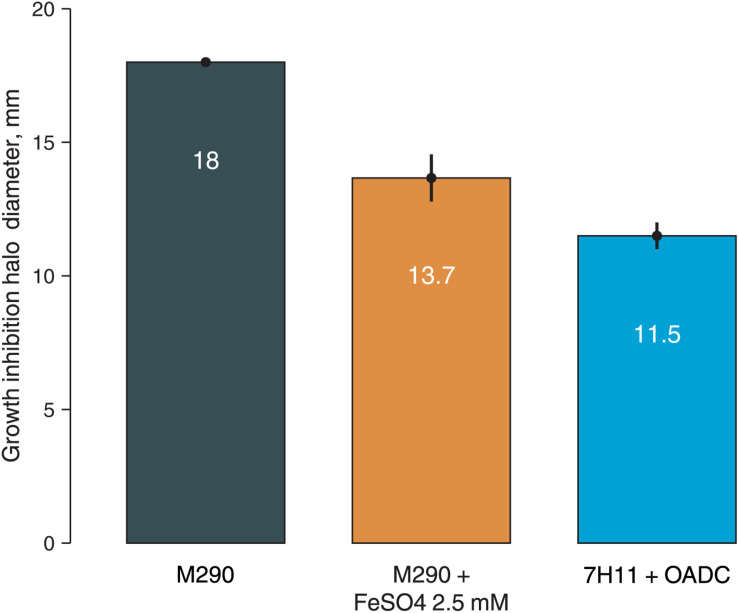
Growth inhibition halos, produced by **3a** (300 nmol/disk) on *M. smegmatis mc2 155* on different media: soyabean digest agar (M290), soyabean digest agar + 2.5 mM FeSO_4_, and 7H11 + OADC. Columns represent the average growth inhibition halo diameters, while the error bars represent the standard deviation, calculated from three experiments.

To prove this hypothesis, we have additionally tested *M. smegmatis* susceptibility to **3a** by paper disk method on solid media with different iron content: soyabean digest agar (no iron in the composition), soyabean digest agar with 2.5 mM FeSO_4_, and Middlebrook 7H11 + OADC medium (containing 40 mg/l ferric ammonium citrate, ∼150 μM). *M. smegmatis* showed higher resistance on both iron-supplemented media as compared to the iron-free soyabean digest agar, confirming that the addition of iron reduces the susceptibility of bacterial cells to **3a**, presumably by reducing the iron starvation (Figure 3).

## Discussion

The transcriptomic analysis of *M. smegmatis mc2 155*, exposed to increasing doses of the imidazo[1,2-*b*][1,2,4,5]tetrazine **3a** has revealed a dose-dependent change in the expression of many genes involved in iron metabolism, including the genes responsible for siderophore synthesis and transport: the gene cluster responsible for exochelin synthesis and transport, *mbtA-G* and *mbtK-N* clusters, ESX-3 operon, *irtA-B*, *mmpS5–mmpL5*, and *bfrA-B* operons.

Our observations based on docking studies suggest that imidazo[1,2-*b*][1,2,4,5]tetrazine **3a** could inhibit the iron metabolizing enzymes and may target the proteins involved in mycobactin biosynthesis, but relatively higher affinities were observed with the proteins involved in exochelin biosynthetic pathways. Virtual screening showed that FxuA is the most likely target of **3a**, followed by FxbA, MbtC, mbtD, and FxbC. The inhibition of siderophores’ synthesis by **3a** could lead to the low-iron conditions transcriptomic profile, observed in our study with a few exceptions. This mechanism could also explain a higher sensitivity of *M. tuberculosis* strains to imidazo[1,2-*b*][1,2,4,5]tetrazines (Maslov et al., 2019), as *M. tuberculosis* is more sensitive to iron starvation as a human pathogen (Doherty, 2007; Skaar, 2010), as well as the higher **3a** MIC in 7H9 medium (128 μg/ml), containing ferric ammonium citrate, as compared to the Lemco-Tw broth (64 μg/ml), which contains no additional iron in its composition (Maslov et al., 2019, 2020). As shown in this study, the addition of iron reduced *M. smegmatis* **3a** susceptibility on agarized medium as well.

Exochelin is a siderophore specific to non-virulent mycobacteria (Ratledge and Ewing, 1996). The products of the *mbtA-G* and *mbtK-N* gene clusters are part of a complex mechanism for the synthesis of siderophores in the cell. Mutation of any *mbt* gene can disrupt the synthesis of siderophores, which, in turn, prevents bacteria from assimilating iron from the environment, leading to impaired cell growth. It was shown that the *M. tuberculosis* H37Rv mutant, in which the *mbtB* gene was replaced by recombination with a hygromycin resistance cassette, was restricted for growth in a medium with limited iron but grew normally in a medium with a high iron content (Voss et al., 2000). The ESX-3 operon encodes a type VII secretion system. These systems are common in mycobacteria (Bitter et al., 2009) and represent a complex with many components and many substrates. The function of these systems has not yet been fully studied, however, there is evidence of their involvement in the secretion of virulence factors and host–pathogen interaction in *M. tuberculosis* (Bitter et al., 2009). Both *mbtA-G* genes products and type VII secretion systems are currently considered promising targets for new anti-TB drugs (Bottai et al., 2014; Maganti et al., 2014).

The *mbtA-G*, ESX-3, *mmpS5–mmpL5*, and *irtA-B* are controlled by HupB and iron levels (Pandey et al., 2014b), some of them in IdeR-dependent (*mbtA-G*, *bfrA-B*) and others in the IdeR-independent way (Rodriguez et al., 2002). HupB and IdeR are two key regulators of iron metabolism in mycobacteria (Rodriguez et al., 2002; Pandey et al., 2014b). HupB is a 22 kDa (214 a.a.) protein with a C-terminal region unique to mycobacteria. The orthologs of HupB in mycobacterial species differ by about 20–30% in their amino acid sequence. HupB binds to the 10 bp region (HupB-box) and activates its regulated genes’ expression (Pandey et al., 2014b), while IdeR acts as a transcription repressor: in high-iron conditions, two IdeR proteins form a complex with Fe^2+^ ions and is able to bind to the IdeR-box, preventing the start of transcription (Rodriguez et al., 2002). When iron level is low, the IdeR-Fe^2+^ complex is disrupted, and transcription is enabled. In another study by Pandey et al. (2014a) it has been shown that the promoter region of *hupB* contains 2 IdeR-boxes and a HupB-box, suggesting that *hupB* could also be regulated by HupB/IdeR system (self-upregulated in low-iron conditions). While it has been shown *in vitro*, that HupB and IdeR can both bind to the *hupB* promoter (Pandey et al., 2014a), the upregulation of HupB was not observed in low-iron conditions in other HupB/IdeR-related studies (Rodriguez et al., 2002; Pandey et al., 2014b), meaning that this mechanism is not necessarily functional in the cell culture. We did not observe any *hupB* upregulation as well.

The HupB/IdeR was reported to be regulated by mycobacterial STPKs PknE, PknF, and PknB (Gupta et al., 2014). Our previous studies showed that **3a** could be docked in the PknB adenine-binding socket (Bekker et al., 2012). However, our current docking studies revealed lower affinity of **3a** toward PknE and PknB (−6.8 and −7.3 kcal/mol, respectively) and to PknF homologs in *M. smegmatis* (ranging from −6.3 to −7.3 kcal/mol), meaning that these proteins could be not the primary targets for this compound. The fact that some iron-sensitive, but HupB/IdeR-independent genes, such as the *nuo* and *pqqE–lldD1* operons (Rodriguez et al., 2002) changed their fold expression dose-dependently (Supplementary Table 1), supports the theory of HupB/IdeR-independent mode of action for **3a**.

The MmpS4–MmpL4 and MmpS5–MmpL5 were shown to be crucial for siderophore transport in *M. tuberculosis* (Wells et al., 2013). Our transcriptomic data shows, that the MSMEG_1381–MSMEG_1382 system might play the primary role in siderophore efflux in *M. smegmatis* among the 3 homologs present in the genome. Our failure to obtain spontaneous **3a**-resistant mutants using the *M. smegmatis* Δ*mmp5* strain could be caused by the accumulation of siderophores in the cell, caused by **3a**-induced siderophore synthesis and the inability to provide their efflux. As the siderophores can be toxic to mycobacterial cells at high concentrations (Jones et al., 2014), this could provide a synergistic effect, requiring more than 1 mutation to override it, and thus drastically lowering the frequency of spontaneous drug-resistant mutants’ emergence.

The understanding of a drug mode of action can be crucial in its development, especially in terms of its structure optimization. We have previously described a set of imidazo[1,2-*b*][1,2,4,5]tetrazines as promising anti-TB drug candidates and shown that mutations leading to *mmpS5–mmpL5* operon overexpression provide *M. smegmatis* efflux-mediated resistance to these compounds. We now show, using the transcriptomic analysis of *M. smegmatis* exposed to different doses of imidazo[1,2-*b*][1,2,4,5]tetrazine **3a**, combined with virtual screening approaches, that this compound may be targeting mycobacterial iron-uptake pathways by inhibiting siderophore synthesis.

## Data Availability Statement

The datasets presented in this study can be found in online repositories. The names of the repository/repositories and accession number(s) can be found in the article/Supplementary Material.

## Author Contributions

DM: conceptualization, project administration, and funding acquisition. AV, ES, MS, DB, and DM: formal analysis. AV, MS, DB, and KK: investigation. DM, AC, and VD: resources. AV, ES, MS, KK, and DM: writing–original draft preparation. ES, MS, DB, and DM: writing review and editing. ES, MS, and DB: visualization. VD and AC: supervision. All authors have read and agreed to the published version of the manuscript.

## Conflict of Interest

The authors declare that the research was conducted in the absence of any commercial or financial relationships that could be construed as a potential conflict of interest.

## Publisher’s Note

All claims expressed in this article are solely those of the authors and do not necessarily represent those of their affiliated organizations, or those of the publisher, the editors and the reviewers. Any product that may be evaluated in this article, or claim that may be made by its manufacturer, is not guaranteed or endorsed by the publisher.

## Footnotes

1
https://uniprot.org
