# Supplementary material for: Transcriptomic Profile of Mycobacterium smegmatis in Response to an Imidazo[1,2-b][1,2,4,5]tetrazine Reveals Its Possible Impact on Iron Metabolism
Source: Front Microbiol. 2021 Aug 4;12:724042. doi: 10.3389/fmicb.2021.724042 (PMC8371482; doi:10.3389/fmicb.2021.724042)
Supplement: Supplementary file 1 [file Presentation_1.pdf]

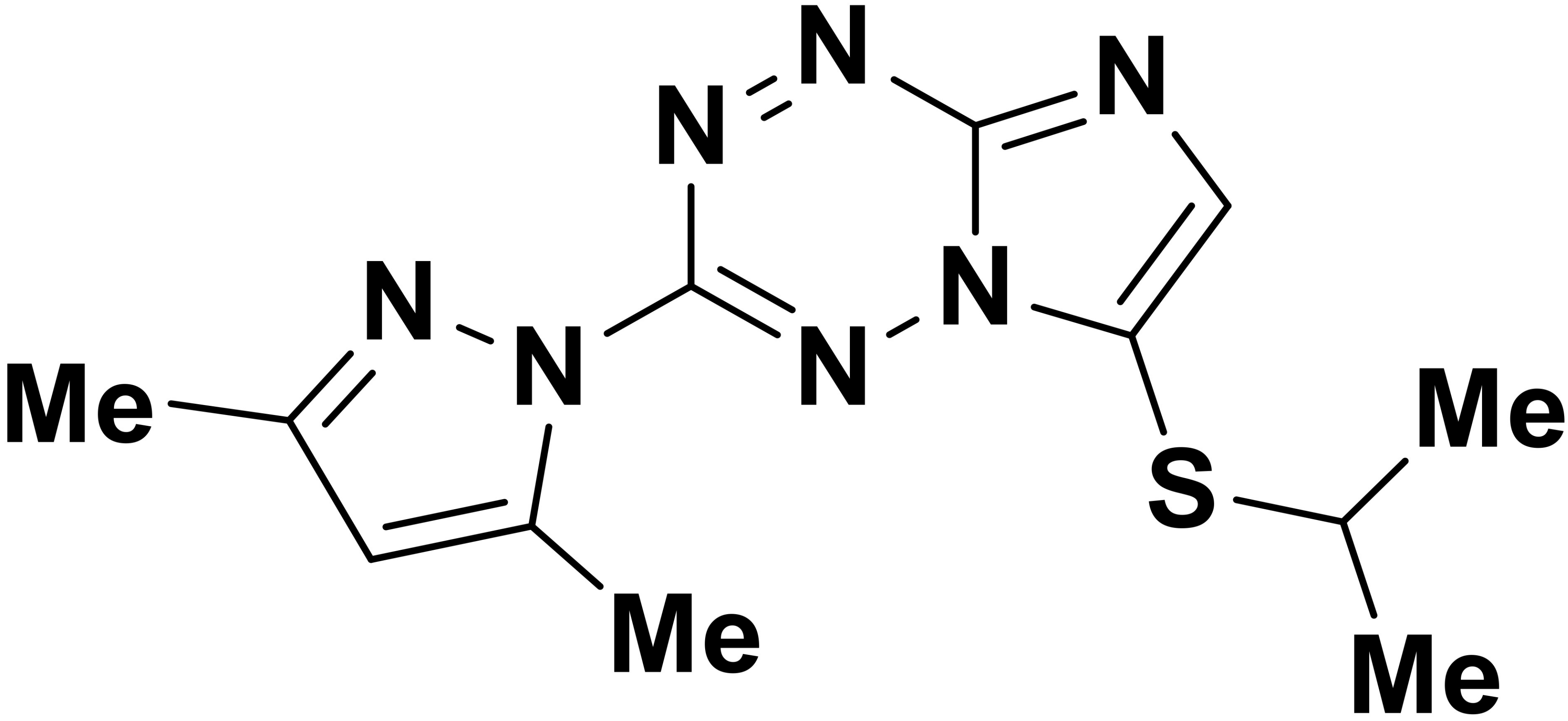


**Figure S1.** Chemical structure of the compound **3a** (Maslov et al., 2019).

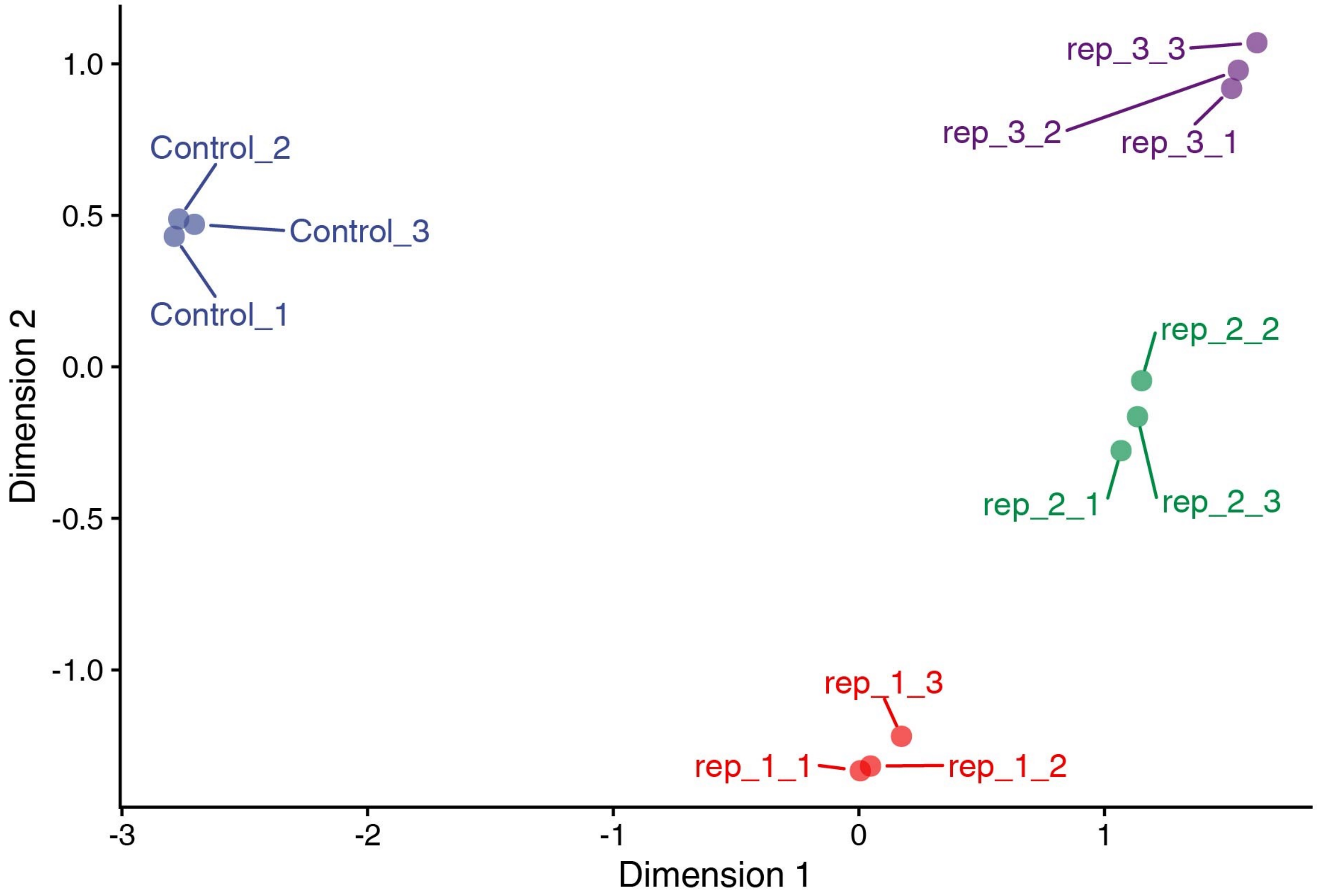


**Figure S2.** Multidimensional scaling plot. Clustering of samples is based on the distances that correspond to the differences in the biological coefficient of variation between the paired samples.

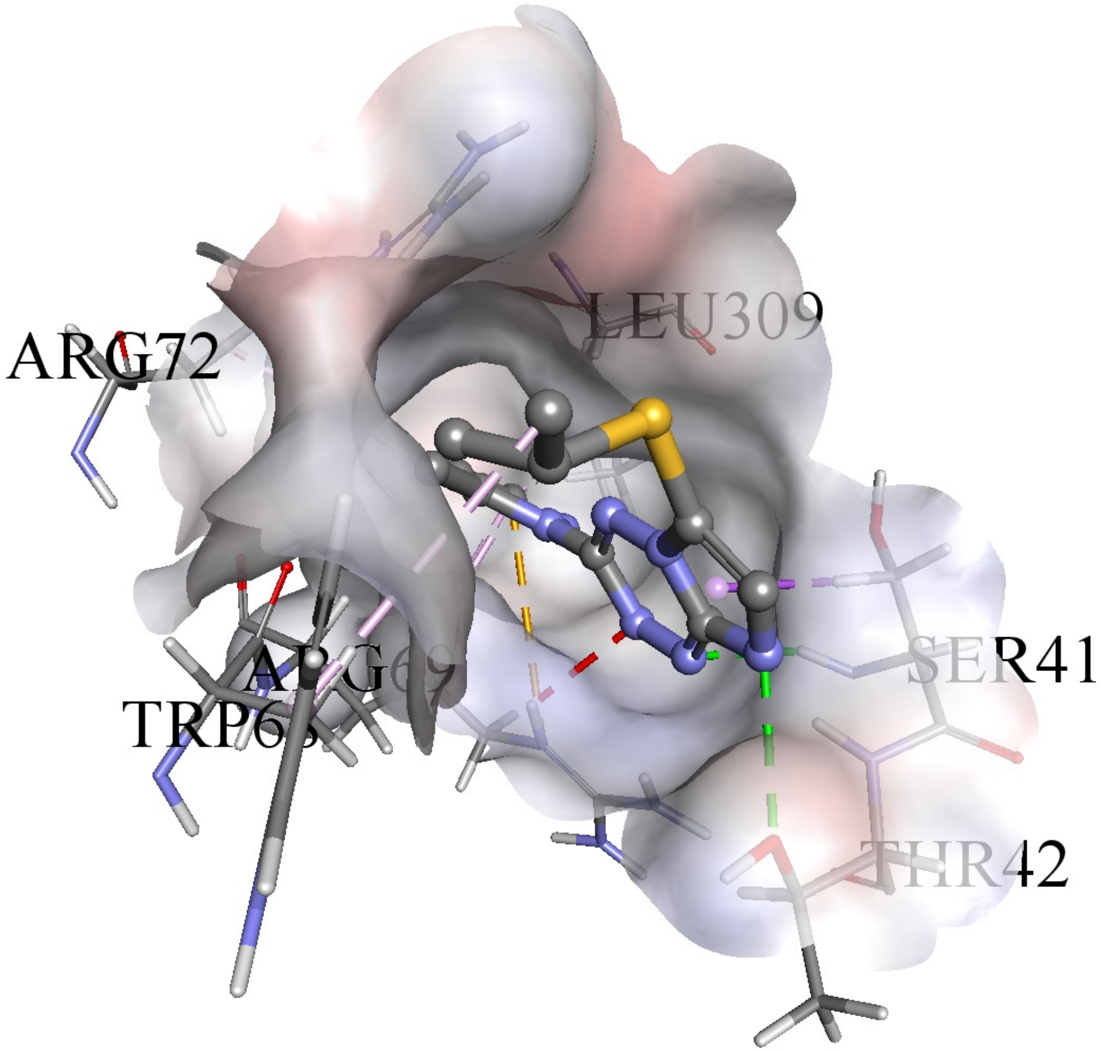


**Figure S3.** Docking of **3a** and FxuA.

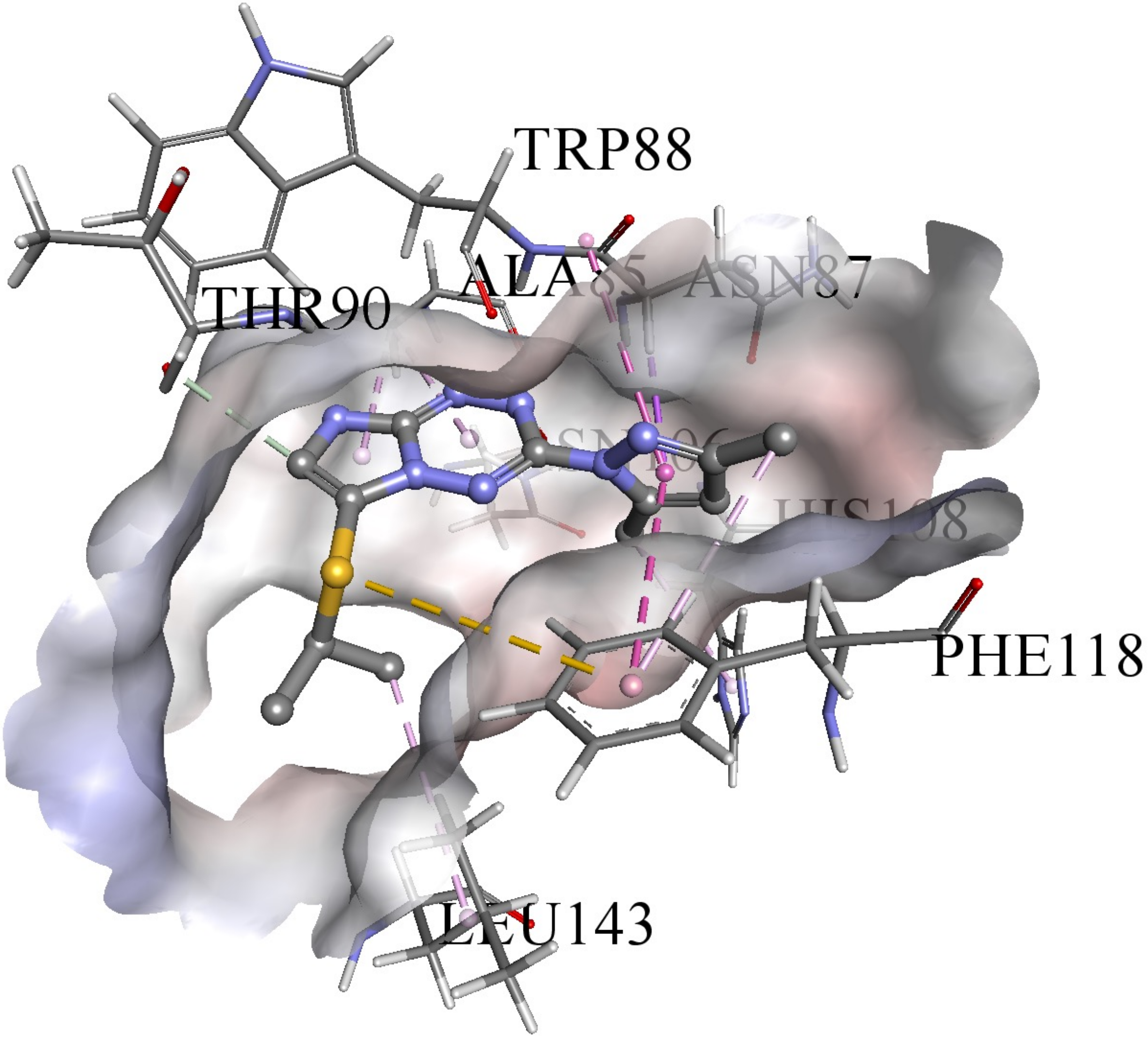


**Figure S4.** Docking of **3a** and FxbA

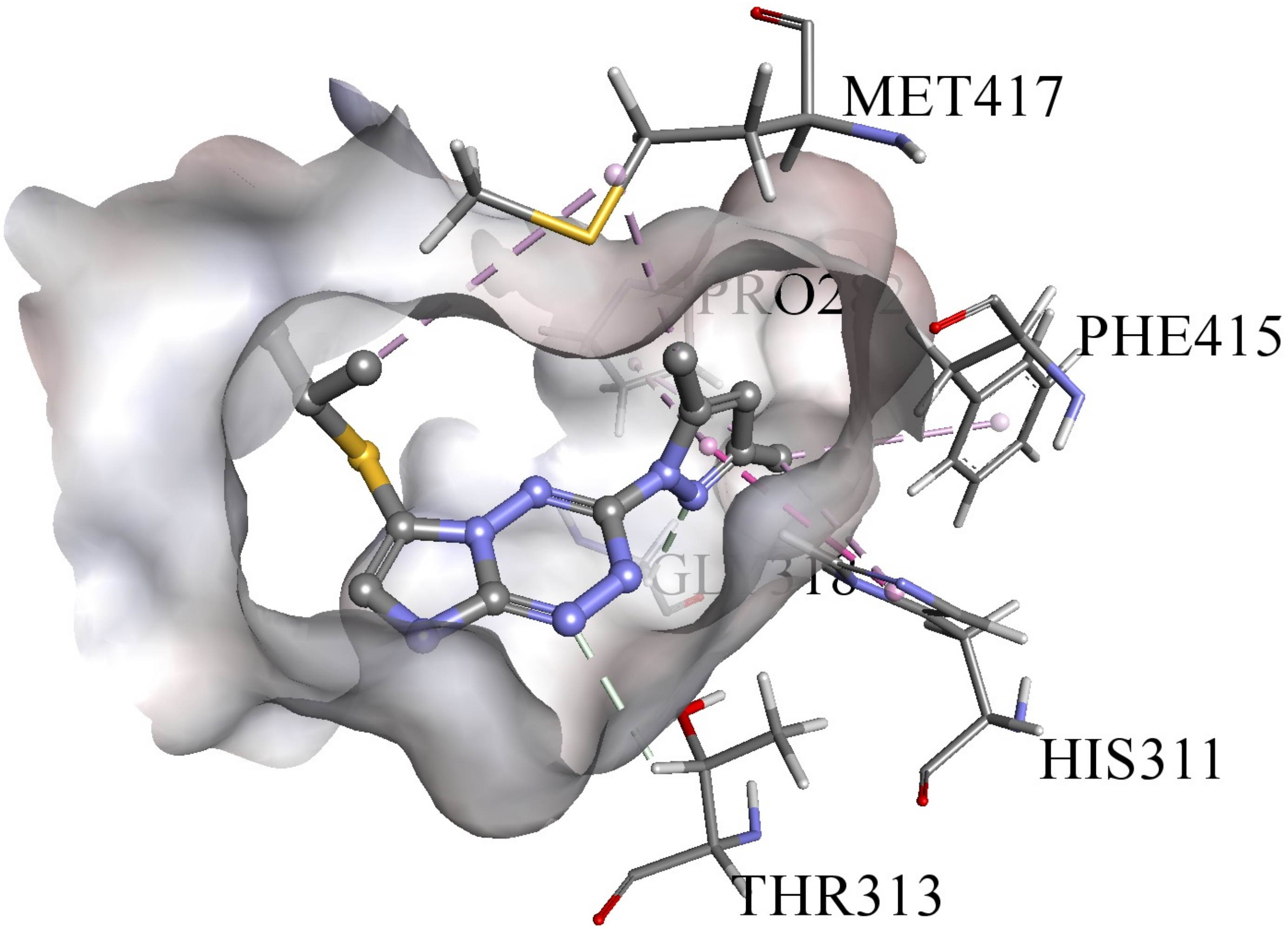


**Figure S5.** Docking of **3a** and MbtC.

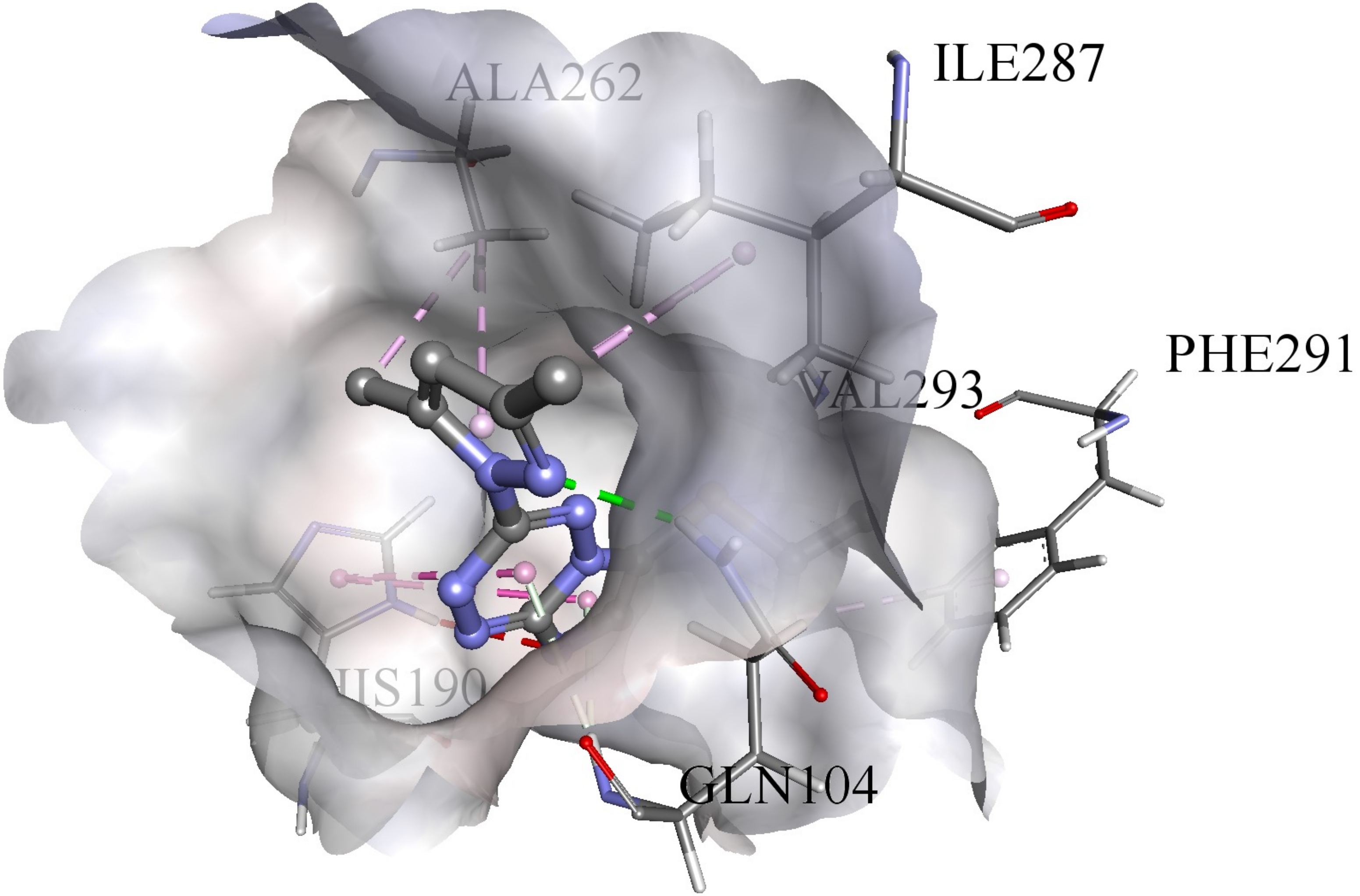


**Figure S6.** Docking of **3a** and MbtD.

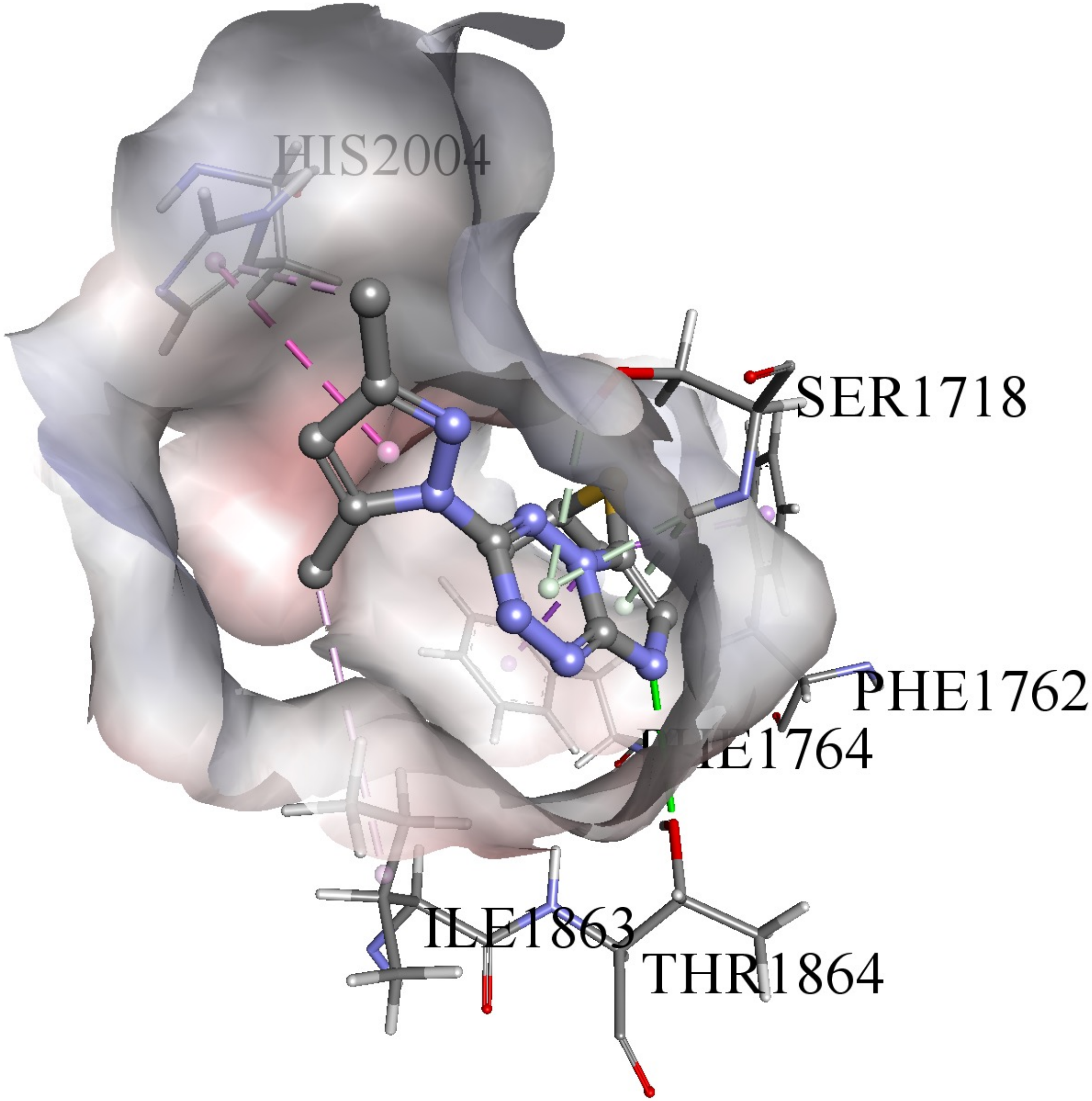


**Figure S7.** Docking of **3a** and FxbC.

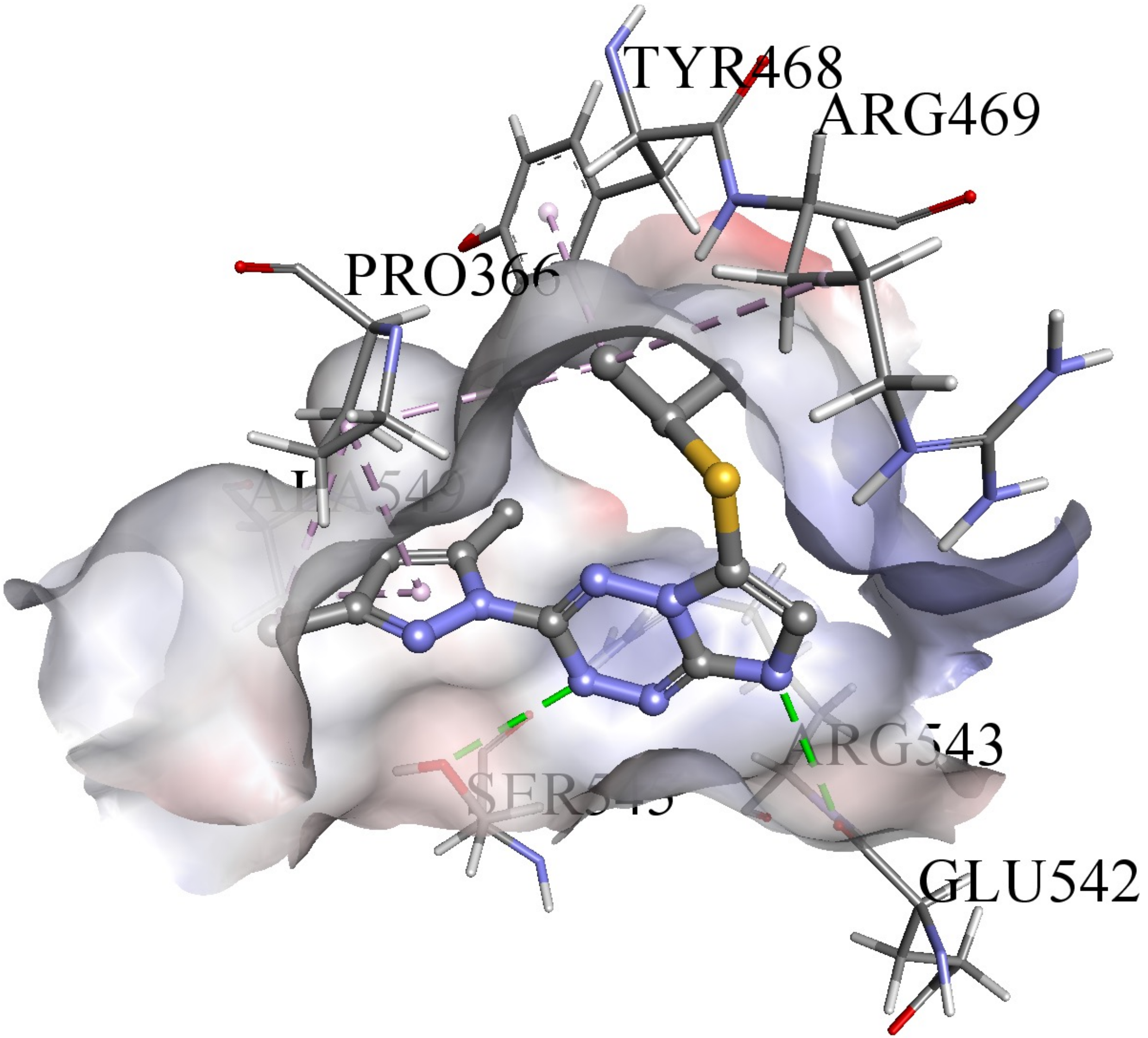


**Figure S8.** Docking of **3a** and EccA3.
